# JAK Inhibition Differentially Affects NK Cell and ILC1 Homeostasis

**DOI:** 10.3389/fimmu.2019.02972

**Published:** 2019-12-19

**Authors:** Laura Vian, Mimi T. Le, Nathalia Gazaniga, Jacqueline Kieltyka, Christine Liu, Giuseppe Pietropaolo, Stefania Dell'Orso, Stephen R. Brooks, Yasuko Furumoto, Craig J. Thomas, John J. O'Shea, Giuseppe Sciumè, Massimo Gadina

**Affiliations:** ^1^Translational Immunology Section, Office of Science and Technology, National Institute of Arthritis Musculoskeletal and Skin Diseases, National Institutes of Health, Bethesda, MD, United States; ^2^Laboratory Affiliated to Istituto Pasteur Italia-Fondazione Cenci Bolognetti, Department of Molecular Medicine, Sapienza University of Rome, Rome, Italy; ^3^Genomic Technology Section, Office of Science and Technology, National Institute of Arthritis Musculoskeletal and Skin Diseases, National Institutes of Health, Bethesda, MD, United States; ^4^Biodata Mining and Discovery Section, Office of Science and Technology, National Institute of Arthritis Musculoskeletal and Skin Diseases, National Institutes of Health, Bethesda, MD, United States; ^5^Division of Preclinical Innovation, National Center for Advancing Translational Sciences, National Institutes of Health, Bethesda, MD, United States; ^6^Lymphoid Malignancies Branch, National Cancer Institute, National Institutes of Health, Bethesda, MD, United States; ^7^Molecular Immunology and Inflammation Branch, National Institute of Arthritis, and Musculoskeletal and Skin Diseases, National Institutes of Health, Bethesda, MD, United States

**Keywords:** JAK/STAT, cytokines, NK cells, ILC, differentiation, kinase inhibitors

## Abstract

Janus kinase (JAK) inhibitors are widely used in the treatment of multiple autoimmune and inflammatory diseases. Immunologic and transcriptomic profiling have revealed major alterations on natural killer (NK) cell homeostasis associated with JAK inhibitions, while information on other innate lymphoid cells (ILCs) is still lacking. Herein, we observed that, in mice, the homeostatic pool of liver ILC1 was less affected by JAK inhibitors compared to the pool of NK cells present in the liver, spleen and bone marrow. JAK inhibition had overlapping effects on the transcriptome of both subsets, mainly affecting genes regulating cell cycle and apoptosis. However, the differential impact of JAK inhibition was linked to the high levels of the antiapoptotic gene Bcl2 expressed by ILC1. Our findings provide mechanistic explanations for the effects of JAK inhibitors on NK cells and ILC1 which could be of major clinically relevance.

## Highlights

- JAK inhibition has distinct impacts on the homeostatic numbers of NK cells and ILC1.
- Tofacitinib has redundant effects on the transcriptomic programs of NK cells and ILC1.
- Basal expression level of Bcl2 underlies the differential impact of tofacitinib in NK cells and ILC1.

## Introduction

Cytokines are pivotal in the maintenance of an appropriate immune system homeostasis, but dysregulation of their activity underlies multiple immune-related disorders (1). The elucidation of the role of the Janus kinase (JAK) family of intracellular tyrosine kinases in the signaling cascade downstream of cytokine receptors has highlighted this class of molecules as potential therapeutic targets. Indeed, inhibition of JAK enzymatic activity has proved successful for several immune-mediated pathologies and these drugs are now approved and prescribed to thousands of patients around the world (2). Given the clinical relevance of the drugs that target these enzymes, more complete knowledge is clearly needed. As such, the pharmacological manipulation of JAKs represents an interesting strategy to study the homeostatic requirements of cytokine signals in different immune cell types ranging from T and B cells to innate lymphoid cells (ILCs).

ILCs provide rapid immune protection through an array of effector functions mirroring those associated with T cells (3). Based on this similarity, ILCs have been divided into five prototypical subsets: natural killer (NK) cells, ILC1, ILC2, ILC3, and lymphoid tissue inducer cells (3). NK cells and ILC1 are able to quickly release the signature cytokine interferon (IFN)-γ and, for this reason, were initially included within the group of type-1 ILCs (4, 5). The ontogeny of ILC1 and NK cells is thought to have both overlapping and independent routes (6, 7); among the latter, evidence in mice shows a distinct usage of T-box transcription factors with a selective expression and function for Eomes in NK cells in contrast to a specific requirement for T-bet in ILC1 (8).

In the context of ILC biology, JAK inhibitors (JAKinibs) have been employed to track different functional outputs, including cytokine production and cell proliferation upon cytokine stimulation *in vitro* (9). Notably, when used *in vivo*, JAKinibs have led to a reduction of the number of mouse NK cells (10). Likewise, patients treated with JAKinibs display a dose-dependent loss of peripheral blood NK cells (11–13). However, no information is currently available about the effects of *in vivo* treatment of JAKinibs on the phenotype of NK cells or other ILCs in distinct tissues.

Development and homeostasis of both NK cells and ILC1 depend on the functions of cytokines, primarily IL-15 and IL-7, which signal through the JAK/STAT pathway (14–16). Observations in humans, corroborated by studies using animal models, have shed light on the importance of the downstream signaling events induced upon activation of JAK3, JAK1, and STAT5 in the development and effector functions of ILCs (17). In this regard, patients carrying *JAK3* mutations develop severe combined immunodeficiency associated with loss of T and NK cells as well as the entire ILC system (18, 19). In mice, *Jak3* deficiency blocks NK/ILC differentiation in the bone marrow (BM) at the ILC precursor and the pre-NK cell progenitor stage; thus, no ILCs are preserved in these mice (20). Similarly, ablation of both *Stat5a* and *Stat5b* leads to almost total loss of NK cells (21). This phenotype is also observed when the entire *Stat5* locus or *Jak1* are deleted in *Ncr1*-expressing cells (22, 23). Selective preservation of *Stat5* alleles (*Stat5b* or *Stat5a*) has revealed a critical role of *Stat5b* more so than *Stat5a* in regulating ILC functions (24, 25), as well as a differential susceptibility among ILCs to tolerate deprivation of STAT5 signals, with NK cells and ILC1 being the most sensitive (25). The profound effects on lymphoid development leading to loss of ILC populations reveal a major limitation in using *Jak3, Jak1*, and *Stat5* deficient mice. Because many of the downstream effects of the JAK/STAT pathway affect the functions of the immune system, distinct compounds capable of blocking JAK enzymatic activity have been developed as selective immunosuppressant to be used in immune-mediated diseases (26).

Herein, we studied the impact of JAKinibs on the homeostasis of two prototypical ILC subsets: NK cells and ILC1. We assessed the effects of *in vivo* administration of a JAK1/3 inhibitor, tofacitinib, vs. a more selective JAK3 inhibitor, PF-06651600, focusing on NK cells from spleen, liver and BM and ILC1 from liver. Our data revealed differential effects of these JAKinibs on the NK cell and ILC1 numbers, the latter subset being less sensitive to JAK inhibition. By using a transcriptomic approach, we identified a major cell cycle block in both subsets after *in vivo* treatment with tofacitinib, associated with a decreased expression of antiapoptotic genes, including *Bcl2*. By using a pharmacological approach, we demonstrated that the high expression levels of *Bcl2* in ILC1 were associated with the differential impact of JAK inhibition observed between the two subsets, arguing for divergent dependence of the homeostasis of these populations on cytokine signals.

## Materials and Methods

### Mice and Inhibitors

BALB/c and *Rag2*^−/−^ mice were purchased from Jackson Laboratory. All animal studies were performed according to NIH guidelines for the use and care of live animals and were approved by the NIAMS Institutional Animal Care and Use Committee. JAKinibs were resuspended in 0.5% methyl cellulose and animals were dosed orally twice daily with vehicle or 30 mg/kg of tofacitinib (kindly provided by Pfizer) or 20 mg/kg of PF-06651600 (provided by the National Center for Advancing Translational Sciences (NCATS), NIH) for 1 week (or 3 days, where indicated) (27). ABT-199 (Venetoclax, Selleckchem) was resuspended in 60% Phosal 50PG, 30% PEG 400, and 10% EtOH. Animals were dosed orally once a day with vehicle or 90 mg/kg for a week.

### Cell Isolation, Flow Cytometry, and Cell Activation Assays

Cells from spleen, liver and BM were isolated as previously described (28). Antibodies are listed in Supplemental Figure 1. Samples were acquired using LSR Fortessa cytometer (BD Biosciences) and BD FACSDiva software (v.8.0.1, BD Biosciences) and analyzed with FlowJo software (Tree Star). Cell sorting was performed using FACSAria III (BD Biosciences). For the evaluation of IFN-γ expression, cells were left untreated or stimulated with PMA/Ionomycin (Sigma-Aldrich) for 2 h or IL-2 (1,000 U/ml, Hoffmann-La Roche Inc.) and IL-12 (10 ng/ml), or IL-12 (10 ng/ml) and IL-18 (100 ng/ml) (R&D Systems) for 6 h (with the addition of GolgiPlug, from BD Biosciences).

### RNA Sequencing and Transcriptomic Analysis

Cells isolated from spleen and liver were sorted (95–99% post-sort purity) as described in Supplemental Figures 1A,B. RNA-seq was performed according to manufacturer's protocol (NEBNext Ultra II RNA Library Prep, E7770L). Barcoded sequencing libraries were sequenced on Illumina HiSeq3000. 50-bp single end reads were demultiplexed to FastQ using bcl2fastq 2.17.1 and mapped onto mouse genome build mm10 using TopHat 2.1.1. Gene expression values (RPKM, reads per kilobase exon per million mapped reads) were calculated with Partek Genomic Suites 7.18.0723. RPKM values were log2 transformed (with a 0.1 offset) and ANOVA was performed to find differentially expressed genes. Expressed genes having an average absolute RPKM > 2 were listed in Supplemental Tables 1, 2 for NK and ILC1, respectively (*miRs* and *Snors* were excluded) and used for further analyses. Volcano plots were generated using R 3.6.0; heatmaps were generated using Morpheus software (Broad Institute). DAVID bioinformatics resource was used for GO analysis.

### Statistics

Unpaired *t*-test and ANOVA were used to quantify statistical deviation between experimental groups, as indicated in figure legends. Asterisks denote significant differences ^*^P < 0.05; ^**^P < 0.01; ^***^P < 0.001.

## Results

### Distinct Impact of JAK Inhibition on ILC1 and NK Cell Homeostatic Numbers

Immunologic and transcriptomic analysis performed on a wide range of adaptive and innate immune cells in mice have revealed a major impact of JAKinibs on the homeostatic pool of splenic NK cells (10). Building on these findings, we sought to dissect how prototypical liver ILC1 were affected by JAKinibs in relations to NK cells present in the liver, spleen and BM.

**Figure 1:**
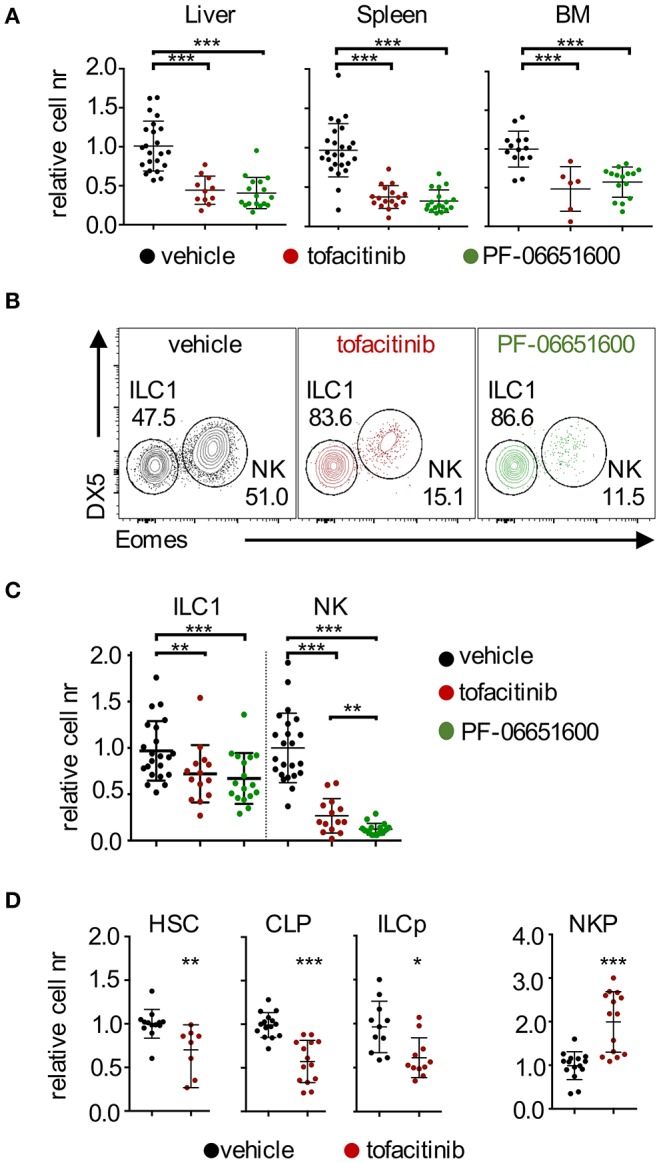
Effects of tofacitinib and PF-06651600 on ILC1, NK cells, and BM progenitors. BALB/c mice were dosed orally with tofacitinib, PF-06651600 or vehicle twice a day for 7 days. **(A)** Relative cell numbers for CD3ε^−^ NKp46^+^ cells from liver (left panel), spleen (middle panel) and BM (right panel) are shown. ANOVA one-way test was applied. **(B)** Subsets of liver NKp46^+^ cells were distinguished by the expression of Eomes and DX5/CD49b. ILC1 were defined as DX5^−^Eomes^−^ cells and NK cells as DX5^+^Eomes^+^ cells. Percentages depicted in dot plots are representative. **(C)** Relative cell numbers for liver ILC1 and NK cells are shown. ANOVA one-way test was applied. **(A–C)** Five independent experiments were combined, and values were normalized to the mean of vehicle-treated mice in the corresponding experiment. **(D)** Relative cell numbers of HSC, CLP, ILCp, and NKP progenitors are shown. Student's *t*-test statistics are comparing samples to vehicle. Three independent experiments were combined, and values were normalized to the mean of vehicle-treated mice for each corresponding experiment. *P < 0.05; **P < 0.01; ***P < 0.001.

We used, as a model, mice treated with oral administration of a JAK1/3 or JAK3/TEC family (29) kinase-selective inhibitors, tofacitinib and PF-06651600, respectively, for a week, twice daily at doses comparable to the range approved for clinical use and which do not provide a total block of JAK3/1 activity (10). We analyzed lymphocytes isolated from liver, spleen and BM by flow cytometry and assessed the relative number of NKp46^+^ cells (gating strategies in Supplemental Figure 1A). Treatment with both JAKinibs led to a marked and significant reduction of the number (represented as ratio relative to control) of NKp46^+^ cells in all tissues analyzed (Figure 1A). Whereas, splenic and BM NKp46^+^ cells mainly comprise NK cells, the liver contains similar proportions of tissue resident ILC1 and NK cells. When we dissected liver NKp46^+^ cells by CD49b (DX5) and Eomes expression, we observed profound and significant changes of NK/ILC1 ratios (Figure 1B). This phenotype was associated with a differential effect in maintaining the homeostatic pools of ILC1 and NK cells. Indeed, while both NK cell and ILC1 numbers were reduced, NK cells were affected to a greater degree than ILC1 (Figure 1C and Supplemental Figure 1B). The differential impact of JAK inhibition on NK cells and ILC1 was independent by the presence of T and B lymphocytes, since similar results were obtained in *Rag2*^−/−^ mice (Supplemental Figure 1C). Moreover, while evidence proving a similar efficacy of inhibiting JAK3 alone or both JAK1 and JAK3 has remained controversial (10, 30), our results showed that selectively targeting JAK3 (and TEC kinases), with PF-06651600, was as efficient as targeting multiple JAKs using tofacitinib in terms of their impact on the homeostatic pools of NKp46^+^ cells.

Together with the effects in liver and spleen, our data provided evidence for the impact of JAKinibs on the pool of BM NK cells (Figure 1A), which led us to evaluate whether JAK inhibition affected NK cell/ILC progenitors present in this tissue. As shown in Figure 1D (gating strategies in Supplemental Figure 1D), treatment with tofacitinib resulted in decreased numbers of HSC, CLP, and ILCp, as well as an accumulation of NKP, pointing to a developmental block at this stage. These findings were in agreement with a previous report showing that selective ablation of the *Stat5* locus in NK cells led to increased numbers of NKP (22).

Altogether, our data showed that administration of JAKinibs at doses within the range approved for clinical use, affected the homeostatic pool of both ILC1 and NK cells, although the impact of JAK inhibition was greater on NK cells. This effect was independent from the presence of adaptive immune cells. In addition, we showed that the effects of tofacitinib extended to BM precursors and ILC/NK progenitors.

### Tofacitinib Administration Inhibits the Expression of Genes Regulating Cell Cycle and Survival in NK Cells

We have previously shown that NK cells with reduction in *Stat5b* or *Stat5a* levels exhibit a loss of their signature traits associated with a maturation block (25). Therefore, we investigated whether treatment with JAKinibs could also affect NK cell identity and/or differentiation by coupling transcriptomic analysis and flow cytometry.

**Figure 2:**
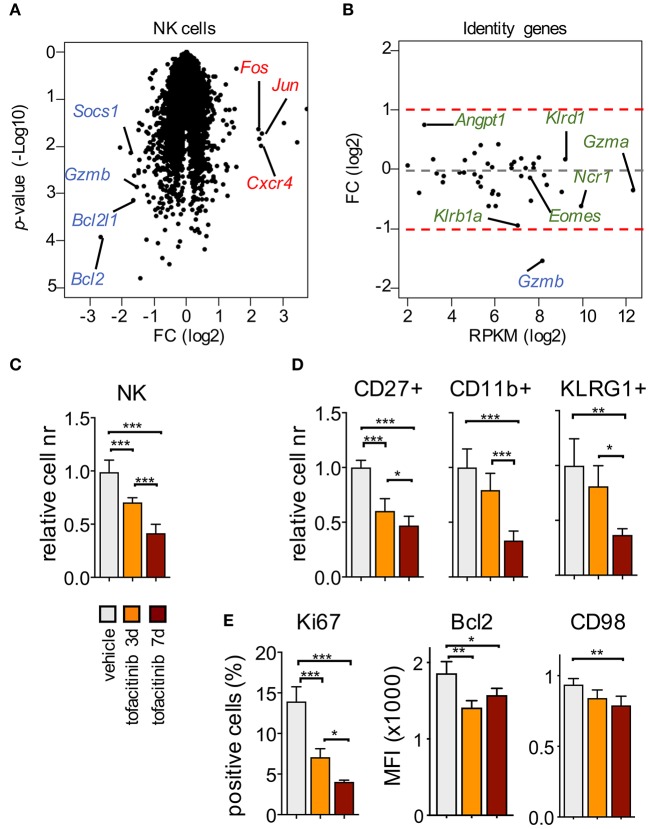
Impact of tofacitinib on the transcriptome and phenotype of splenic NK cells. **(A)** Volcano plot for genes expressed by splenic NK cells isolated from mice treated or not for 1 week with tofacitinib. Representative down-regulated (blue) and up-regulated (red) genes are highlighted. Two mice for treated and three for vehicle were pooled together for each replicate. **(B)** MA plot shows transcript abundance (x axis, mean RPKM in NK cells receiving vehicle) and FC (y axis, log2 of tofacitinib ÷ vehicle) for genes associated with NK cell identity. **(C)** Relative cell number for splenic NKp46^+^ and **(D)** NK cell subsets, defined by CD27, CD11b, or KLRG1 expression, at 3 and 7 days of tofacitinib treatment are shown. **(E)** Expression levels evaluated by flow cytometry for Ki67 (percentage of positive cells), Bcl2 [Mean Fluorescence Intensity (MFI)], and CD98 (MFI) in NK cells at 3 and 7 days of tofacitinib treatment are shown. **(C–E)** Statistics were performed using one-way ANOVA. Two independent experiments were combined (vehicle *n* = 8; 3-days treatment *n* = 5; 7-days treatment *n* = 6), and values were normalized to the mean of vehicle-treated mice for each corresponding experiment. *P < 0.05; **P < 0.01; ***P < 0.001.

At the transcriptional level, we observed that the impact of the 7-days treatment with tofacitinib mainly consisted of a reduction in gene expression in splenic NK cells (Figure 2A and Supplemental Table 1), which included down-regulation of genes involved in NK cell survival (*Bcl2* and *Bcl2l1*), proliferation (*Mki67*), and function (*Gzmb*), in agreement with a previous report (10). On the other hand, genes defining NK cell identity [gene list described by the Immgen project (31)] were not affected by the treatment, except for *Gzmb*, which was the only down-regulated gene with a fold-change (FC) higher than 2 (Figure 2B). Similarly, no significant differences in *Eomes* and *Tbx21* (encoding T-bet) were observed after *in vivo* treatment with tofacitinib.

To evaluate the effect of tofacitinib treatment on terminal differentiation, we measured the number of NK cells expressing markers associated with distinct maturation stages, namely CD27, CD11b, and KLRG1 (32, 33). To rule out the effects of possible mechanisms of cell adaptation or selection which could occur after the 7-days treatment, mice also received the drug for only 3 days. As shown in Figure 2C, NK cell numbers already started to decrease at the early time point, and to a greater extent at day 7. This reduction was associated with a global alteration of all the NK cell subsets analyzed (Figure 2D), and led, after 7 days of treatment, to a selective decrease of the frequency of terminally differentiated NK cells expressing KLRG1 (Supplemental Figure 2), suggesting that JAK inhibition could have cumulative effects during time either on differentiation or turn-over of this subset.

Finally, we evaluated by flow cytometry the expression levels of selected downregulated genes present in our dataset (Supplemental Table 1), including Ki67 (encoded by *Mki67*) to track cell cycle, Bcl2 for survival and CD98 (as a surrogate for Slc7a5 expression). As shown in Figure 2E, expression of these proteins was significantly affected both at day 3 and 7 after tofacitinib treatment. Among all the parameters analyzed, only Bcl2 was downregulated at higher degree at day 3 than day 7, suggesting that the effect on survival could occur earlier than the effects on proliferation and differentiation.

Our results showed that, in contrast to the previously employed genetic models, acute pharmacological inhibition did not alter NK cell identity suggesting differential requirements for acquisition of signature genes vs. homeostasis. Moreover, except for Bcl2, the impact of JAK inhibition on NK cells appeared to be cumulative during the time frame analyzed and encompassed effects on cell cycle as well as survival.

### Redundant Effects of Tofacitinib on the Transcriptional States and Functions of ILC1 and NK Cells

**Figure 3:**
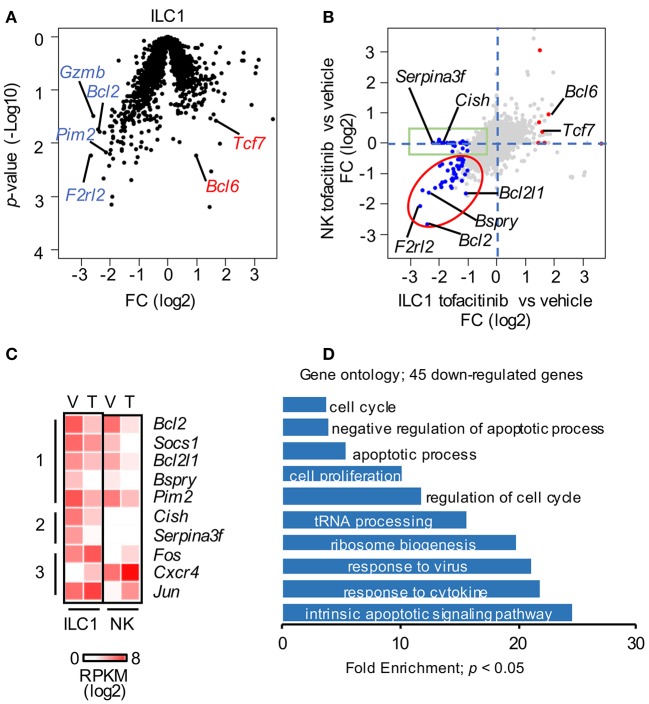
Redundant effect of tofacitinib on ILC1 and NK cell transcriptomes. **(A)** Volcano plot for genes expressed by liver ILC1 isolated from mice treated or not for 1 week with tofacitinib. Representative down-regulated (blue) and up-regulated (red) genes are highlighted. **(B)** Scatter plot comparing the effects of tofacitinib on NK and ILC1. Genes significantly (*p* < 0.05) up-regulated (FC > 2) and down-regulated (FC < 0.5) in ILC1 are highlighted in red and blue, respectively. **(C)** Expression of selected genes in liver and splenic NK cells is depicted by heatmap, comparing mice administered with vehicle or tofacitinib. **(D)** Forty-five down-regulated genes in ILC1 having RPKM > 5; FC < 0.5; *p*-value < 0.05 were selected for GO. Only GO terms with a *p*-value < 0.05 are represented. More than 10 mice for each group (vehicle and tofacitinib) were pooled.

To discriminate possible mechanisms underlying the differential sensitivity to JAKinibs on the homeostatic pool of liver ILC1 and NK cells, we explored the impact of tofacitinib on the transcriptome of ILC1 (Supplemental Table 2). Similar to what observed in NK cells, transcriptomic changes in ILC1 mainly consisted of a reduction in gene expression (Figure 3A, and Supplemental Table 2). Among the few significantly up-regulated genes, only *Tcf7*, a transcription factor expressed by ILC progenitors and required for the development of the whole ILC compartment (34), reached high expression levels of 54 RPKM and a FC higher than 2. Similarly to what we observed in NK cells, genes involved in survival (*Bcl2*), function (*Gzmb*), and proliferation (*Mki67*) were mainly down-regulated in ILC1.

To define distinct and shared genes affected by JAK inhibition on ILC1 and NK cells, we compared the two datasets highlighting the genes up- and down-regulated in ILC1 (FC > 2; *p*-value < 0.05). As shown in Figure 3B, most of the genes down-regulated in ILC1 followed a similar trend in NK cells (red ellipse), including *Bcl2, Bcl2l1*, or *Bspry* (Figure 3C, group 1). Moreover, we observed a discrete fraction of genes that was selectively down-regulated in ILC1 (green box). This group was enriched with genes specifically expressed on ILC1. Among these genes, *Cish*, which encodes for cytokine-inducible SH2 containing protein (CIS), was constitutively expressed on ILC1 and decreased upon *in vivo* treatment with tofacitinib (Figure 3C, group 2). Among the up-regulated genes, *Bcl6* was also induced in NK cells; and vice-versa, genes up-regulated in NK cells, such as, *Fos* and *Jun* followed a similar trend in ILC1 (Figure 3C, group 3).

**Figure 4:**
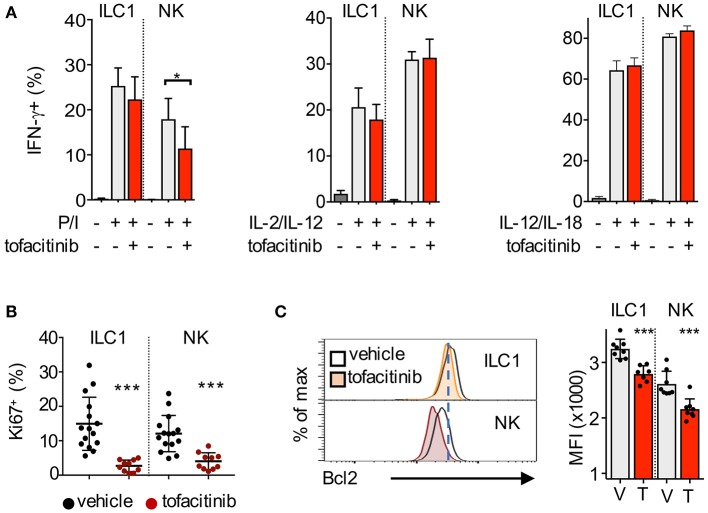
Impact of tofacitinib treatment on the functions of NK cells and ILC1. **(A)** Bar graphs show the percentage of IFN-γ-positive ILC1 and NK cells, quantified by flow cytometry, after 2 h stimulation with PMA/Ionomycin (P/I), or 6 h stimulation with IL-2/IL-12 or IL-12/IL-18, in mice treated or not for 1 week with tofacitinib. Two experiments were combined. **(B)** Percentage of Ki67-positive ILC1 and NK cells isolated from liver after 7 days administration of tofacitinib or vehicle. **(C)** Representative histogram plots (left panel) and bar graphs (MFI, right panel) show Bcl2 protein expression in ILC1 and NK cells from mice treated with tofacitinib or vehicle. *P < 0.05; ***P < 0.001.

Gene ontology (GO) analysis showed that transcripts regulating the response to cytokines/virus, cell cycle and the apoptotic pathway were enriched among the tofacitinib targets in ILC1 (Figure 3D). Thus, we sought to measure whether administration of tofacitinib for 7 days differentially affected the ability to produce IFN-γ, as well as, the expression of Ki67 and Bcl2 in liver ILC1 and NK cells. To evaluate the production of IFN-γ, liver cells were isolated both from untreated and tofacitinib-treated mice and stimulated with PMA/Ionomycin, IL-2/IL-12, or IL-12/IL-18. As shown in Figure 4A, a 7-days treatment with tofacitinib reduced the ability of NK cells to produce IFN-γ upon PMA/Ionomycin stimulation, while the potential of NK cells and ILC1 to respond to cytokines was not altered. These data suggest that the pharmacological block has limited effects on the cell intrinsic abilities to produce IFN-γ (Figure 4A).

Next, we tracked the levels of Ki67 and Bcl2 expression on liver ILC1 and NK cells after 7-days treatment with tofacitinib. Contrary to the impact observed on the homeostatic pools of both subsets, tofacitinib inhibited Ki67 expression at greater degree in ILC1 than in NK cells (Figure 4B). As shown above for splenic NK cells, the effects of tofacitinib on liver ILC1 were already detectable at day 3 and greater at day 7 after treatment (Supplemental Figure 3A). These data provide evidence for the role of JAKinbs in regulating the overall proliferative states of both ILC1 and NK cells, *in vivo*. Relative to Bcl2 expression, we observed that tofacitinib inhibited this protein in both subsets (Figure 4C). However, the expression levels of Bcl2 in liver ILC1 isolated from both untreated and tofacitinib-treated mice exceeded those observed in untreated NK cells, suggesting that the relatively high levels of Bcl2 on ILC1 might be responsible for the limited effect of JAKinibs on the homeostatic pool of these cells.

Altogether, we showed that the effects of JAK inhibition on ILC1 and NK cells appeared redundant at the transcriptional and functional level, with genes involved in survival and proliferation being mainly affected.

### Basal Expression Levels of Bcl2 Are Linked to the Outcome of JAKinibs on the Homeostatic Numbers of ILC1 and NK Cells

The impact of Bcl2 family members in regulating NK cell survival has been previously addressed using genetic models, which have demonstrated that *Bcl2* and *Mcl1* have non-redundant roles in regulating NK cell survival (35–37), while *Bcl2l1* is dispensable (35). In NK cells, Bcl2 is down-regulated when STAT5 signaling is altered (25, 38), and its overexpression can rescue the effects on the NK cell pool associated with *Stat5* deficiency (39). In our dataset, the antiapoptotic genes *Bcl2* and *Bcl2l1* were both downregulated in ILC1 and NK cells, while *Mcl1* expression was not altered by tofacitinib treatment, implying differential mechanisms of homeostatic regulation for these three members of the Bcl2 family.

**Figure 5:**
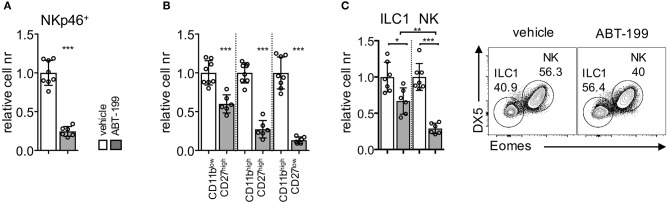
ABT-199 treatment differentially affects the homeostatic pool of liver ILC1 and NK cells. Mice were dosed orally with ABT-199 or vehicle daily for 7 days. **(A)** Relative cell numbers of splenic NK cells and **(B)** NK cell subsets (dissected based on CD27 and CD11b expression) are shown. **(C)** Liver ILC1 and NK cells in mice untreated or treated for 7 days with ABT-199 (Venetoclax) or vehicle are depicted. Two experiments were combined (vehicle *n* = 8; ABT-199 *n* = 6), and values were normalized to the mean of vehicle-treated mice for each corresponding experiment. One-way ANOVA was applied. *P < 0.05; **P < 0.01; ***P < 0.001.

Given that Bcl2 protein was expressed at higher levels by liver ILC1, from both untreated and tofacitinib-treated mice, compared to untreated NK cells, we hypothesized that the differential effect of tofacitinib on the size of the two subsets was dependent on their distinct ability to survive after perturbation of Bcl2 function. To test this hypothesis, we treated mice with oral administration of a Bcl2 specific blocker, namely ABT-199 (Venetoclax) for 7 days (40, 41). We first analyzed whether the pharmacological block of Bcl2 had parallel effects compared with those observed in genetic models by evaluation the impact of ABT-199 on splenic NK cells. As shown in Figure 5A, ABT-199 administration induced a global decrease of the number of splenic NK cells. In addition, treatment mainly affected the number of more differentiated subsets defined according CD27 and CD11b expression (Figure 5B). These observations were in line with the low Bcl2 expression levels observed in mature CD11b^+^ NK cell subsets (Supplemental Figure 3B) and with previous evidence in mice showing major defects in mature NK cells associated with the ablation of *Bcl2* gene.

Having established the impact of ABT-199 administration on splenic NK cells, we next analyzed its effect on the liver subsets. As shown in Figure 5C, treatment with ABT-199 led to a significant decrease of both liver ILC1 and NK cells. However, the number of the NK cell pool was affected at a higher degree in comparison to the number of ILC1, indicating that the higher levels of Bcl2 present on ILC1 provided an advantage in term of survival as compared to NK cells.

Altogether we showed that pharmacological inhibition of Bcl2 in mice recapitulated the effects observed using genetic models targeting Bcl2, consisting in a major loss of more differentiated NK cells. Moreover, we provided evidence for a differential effect of Bcl2 inhibition in ILC1 and NK cells, the latter being more sensitive to ABT-199 treatment.

## Discussion

To better understand the impact of JAK inhibition in regulation of NK cells and ILC homeostasis, we have administered JAKinibs to mice at doses comparable to the range approved for clinical use. The first unexpected finding we observed was the differential impact of JAKinibs on the homeostasis of NK cells and ILC1. This was unanticipated because both subsets were highly affected when *Jak3* and *Stat5* were targeted by genetic approaches in mice (20, 25). Mechanistically, we speculated that the limited effect of JAKinibs on the pool of ILC1 was linked to their higher expression levels of Bcl2 compared to NK cells. This hypothesis is supported by the higher sensitivity of NK cells compared to ILC1, in respect to the pharmacological block of Bcl2.

Along with survival, the cell cycle of both NK cells and ILC1 was highly affected by tofacitinib treatment; the drastic reduction of cells in the G1-S-M phase associated with the low frequency of Ki67^+^ cells outline the pivotal role of JAK signals in regulating the proliferation of these prototypical subsets *in vivo*, and can also contribute to the decrease of their homeostatic number. Interestingly, this aspect, although inferred by results obtained using *in vitro* systems, had remained unexplained by employing genetic approaches, *in vivo*.

The effects observed on the pool of bone marrow NK cells and their precursors represent another possible factor contributing to the to the decreased number of NK cells in other organs. In this regard, tofacitinib treatment induces an increase of the chemokine receptor CXCR4, which could alter mechanisms of bone marrow retention of NK cells (42, 43). Despite the differences on the homeostatic numbers, the effect of JAK inhibition at the transcriptional level was similar for both NK cells and ILC1, with a main reduction of the expression of JAK-targets. No major changes in genes defining the NK cell identity were observed, indicating a differential role for JAK-dependent signals in regulating acquisition of identity and homeostasis. Among the few upregulated genes, we found the transcription factors *Bcl6, Tcf1, Fos*, and *Jun*. These TFs could be usually repressed by JAK-signals or, alternatively, up-regulated after tofacitinib treatment allowing cells to adapt to deprivation of JAK-dependent signals. The expression of Bcl6 has been related to mechanisms of ILC plasticity, involving suppression of ILC3 genes and promotion of NK/ILC1 specific programs (44). Although transitions of NK cells toward an ILC1-like phenotype occur both under physiological and pathological conditions, we did not observe alterations of ILC1 markers, such as Trail and CD49a, in NK cells after treatment (data not shown). Thus, the higher expression of Bcl6 in both NK cells and ILC1 after tofacitinib treatment might be part of a circuit reinforcing NK/ILC1 phenotypes in absence of proper levels of JAK-dependent signals.

While most of the genes followed a common transcriptional trend in NK cells and ILC1 upon tofacitinib treatment, *Cish* represented one of the few exceptions, being differentially targeted by tofacitinib in the two type 1 subsets. As recently reported, the homeostatic expression of *Cish* is very low in NK cells but increases rapidly following IL-15 stimulation (45). Since *Cish* levels were downregulated after *in vivo* treatment with tofacitinib, the constitutive expression of *Cish* in ILC1, instead, could be dependent on the high levels of JAK-dependent signals acting on these tissue-resident cells.

Moreover, we noticed that most of the effects observed in NK cells at day 7 after treatment were also present at an earlier time point. However, the impact of JAK inhibition on NK cells appeared to be cumulative, within the time frame analyzed, in terms of cell numbers, maturation stages and proliferation, with Bcl2 representing the only exception. Thus, our data suggest that the limited effects of tofacitinib on NK cell transcriptome could be independent from mechanisms of adaptation occurring during the treatment, which may select or generate cells resistant to JAK inhibition.

Finally, our study showed that this approach represents both an opportunity to better understand ILC biology as well as a strategy to modulate ILC functions during diseases. Since pharmacological inhibition of JAKs is now successfully utilized for the treatment of several immune-mediated pathologies, our study sheds light on the potential effects on immune cells when this pathway is targeted.

## Data Availability Statement

The datasets generated for this study can be found in the GEO: GSE135116.

## Ethics Statement

The animal study was reviewed and approved by National Institute of Arthritis and Musculoskeletal and Skin Diseases, Institutional Animal Care and Use Committee (IACUC).

## Author Contributions

LV performed the *in vitro, in vivo*, and RNA-seq experiments. ML, NG, CL, JK, GP, SD, and YF helped to perform the experiments. SB processed the transcriptomic data and performed the computational analyses. GS performed the transcriptomic analysis and data visualization. JO'S contributed to the project design, data interpretation, and manuscript writing. CT provided the critical reagents and helped to write the paper. LV, GS, and MG designed the project and wrote the manuscript with input from all authors.

### Conflict of Interest

The National Institutes of Health hold patents on targeting JAKs. National Institute of Arthritis and Musculoskeletal and Skin Diseases (NIAMS) (JO'S and MG) and Pfizer have a Collaborative Research and Development Award (CRADA). The remaining authors declare that the research was conducted in the absence of any commercial or financial relationships that could be construed as a potential conflict of interest.
