# Supplementary material for: JAK Inhibition Differentially Affects NK Cell and ILC1 Homeostasis
Source: Front Immunol. 2019 Dec 19;10:2972. doi: 10.3389/fimmu.2019.02972 (PMC6930870; doi:10.3389/fimmu.2019.02972)
Supplement: Supplementary file 4 [file Presentation_1.PPTX]

## Slide 1
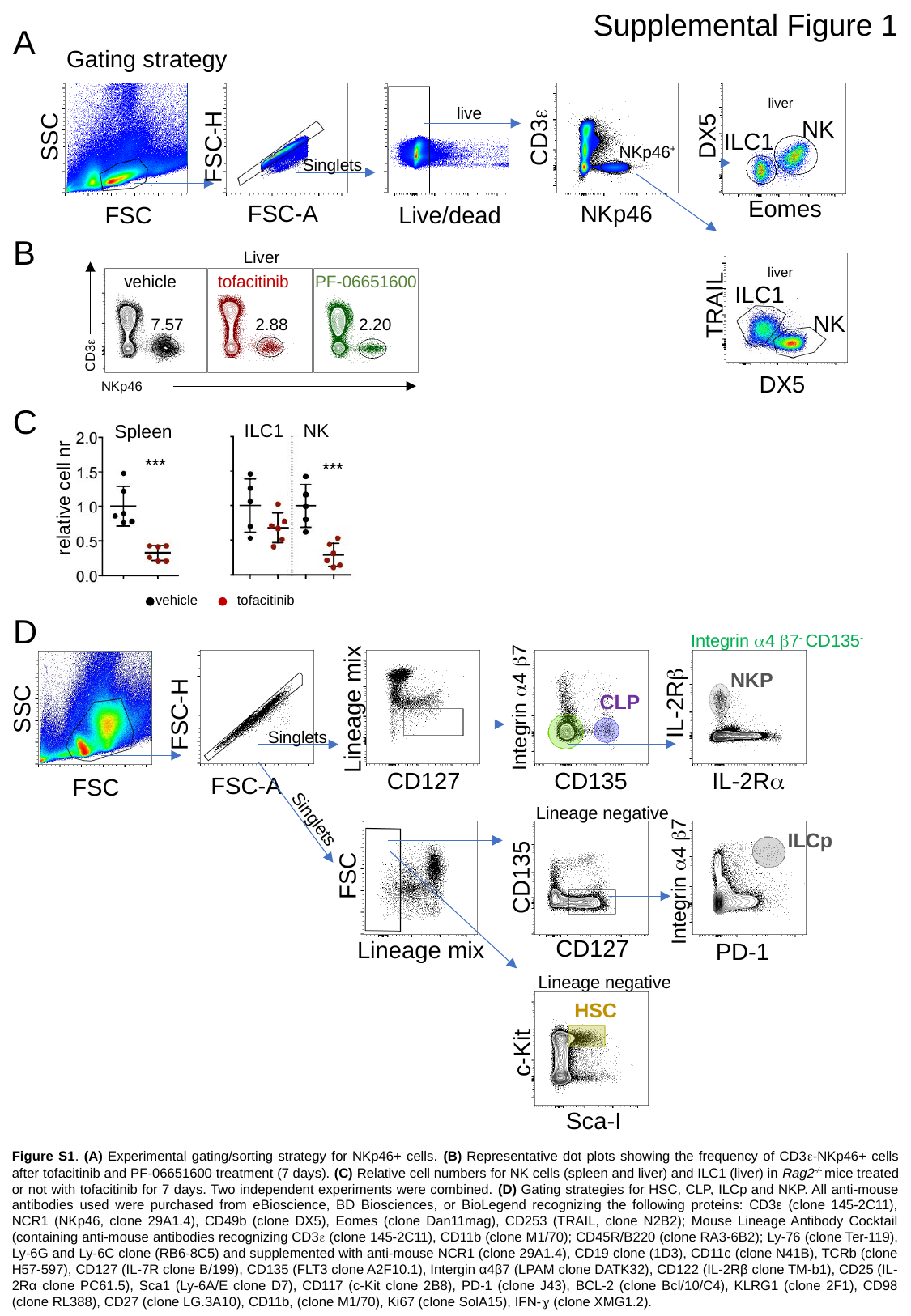

Supplemental Figure 1
A
Gating strategy
liver
live
NK
DX5
CD3e
SSC
ILC1
FSC-H
NKp46+
Singlets
Eomes
NKp46
FSC-A
FSC
Live/dead
B
Liver
liver
7.57
2.88
2.20
CD3e
NKp46
vehicle
tofacitinib
PF-06651600
ILC1
TRAIL
NK
DX5
C
Spleen
***
relative cell nr
ILC1
NK
***
vehicle
tofacitinib
D
Integrin a4 b7- CD135-
NKP
IL-2Rb
CLP
Lineage mix
Integrin a4 b7
SSC
FSC-H
Singlets
CD127
CD135
IL-2Ra
FSC-A
FSC
Lineage negative
Singlets
ILCp
FSC
CD135
Integrin a4 b7
CD127
Lineage mix
PD-1
Lineage negative
HSC
c-Kit
Sca-I
Figure S1. (A) Experimental gating/sorting strategy for NKp46+ cells. (B) Representative dot plots showing the frequency of CD3e-NKp46+ cells after tofacitinib and PF-06651600 treatment (7 days). (C) Relative cell numbers for NK cells (spleen and liver) and ILC1 (liver) in Rag2-/- mice treated or not with tofacitinib for 7 days. Two independent experiments were combined. (D) Gating strategies for HSC, CLP, ILCp and NKP. All anti-mouse antibodies used were purchased from eBioscience, BD Biosciences, or BioLegend recognizing the following proteins: CD3ε (clone 145-2C11), NCR1 (NKp46, clone 29A1.4), CD49b (clone DX5), Eomes (clone Dan11mag), CD253 (TRAIL, clone N2B2); Mouse Lineage Antibody Cocktail (containing anti-mouse antibodies recognizing CD3e (clone 145-2C11), CD11b (clone M1/70); CD45R/B220 (clone RA3-6B2); Ly-76 (clone Ter-119), Ly-6G and Ly-6C clone (RB6-8C5) and supplemented with anti-mouse NCR1 (clone 29A1.4), CD19 clone (1D3), CD11c (clone N41B), TCRb (clone H57-597), CD127 (IL-7R clone B/199), CD135 (FLT3 clone A2F10.1), Intergin α4β7 (LPAM clone DATK32), CD122 (IL-2Rβ clone TM-b1), CD25 (IL-2Rα clone PC61.5), Sca1 (Ly-6A/E clone D7), CD117 (c-Kit clone 2B8), PD-1 (clone J43), BCL-2 (clone Bcl/10/C4), KLRG1 (clone 2F1), CD98 (clone RL388), CD27 (clone LG.3A10), CD11b, (clone M1/70), Ki67 (clone SolA15), IFN-g (clone XMG1.2).

## Slide 2
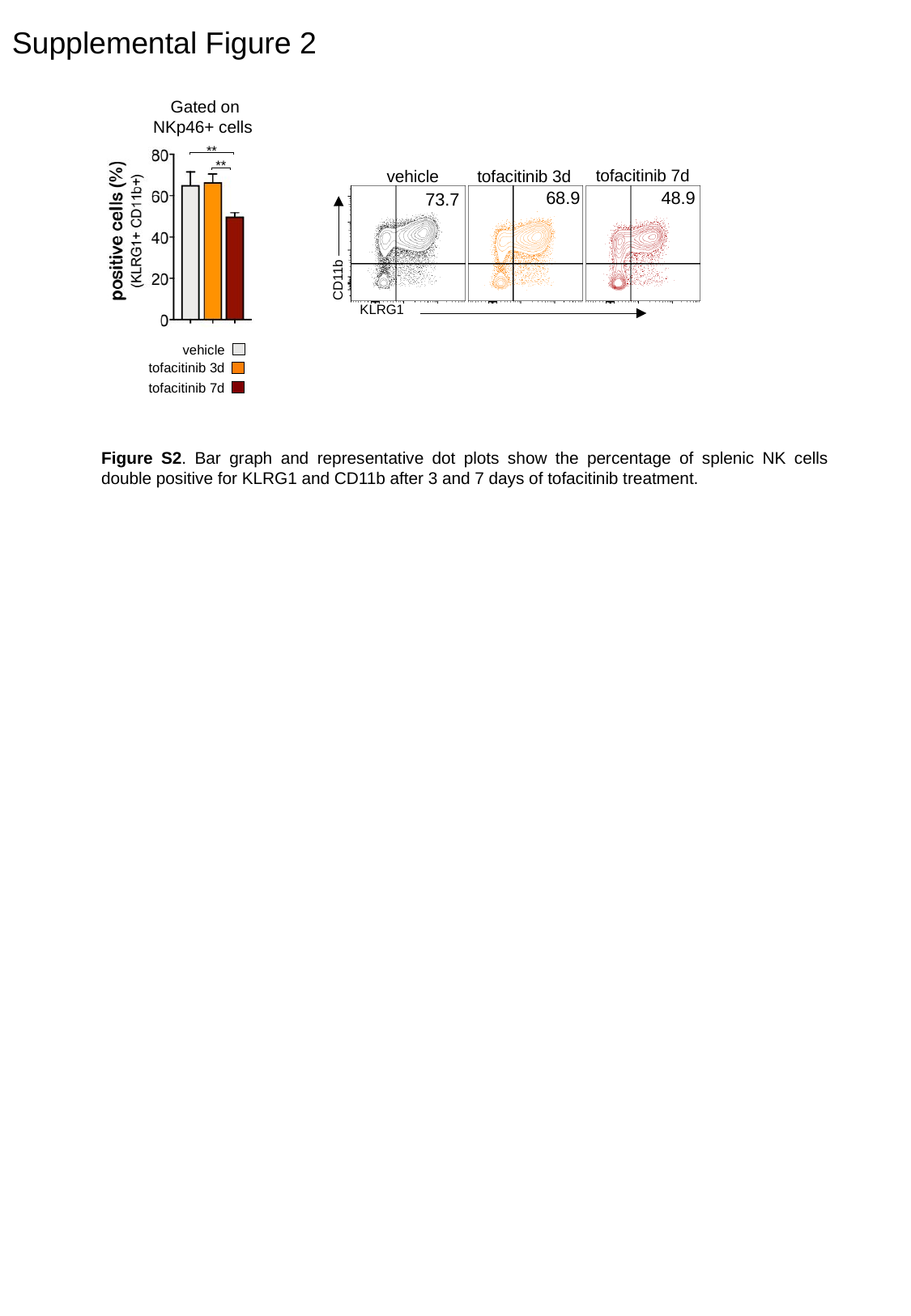

Supplemental Figure 2
Gated on NKp46+ cells
**
**
tofacitinib 7d
vehicle
tofacitinib 3d
68.9
48.9
73.7
CD11b
KLRG1
vehicle
tofacitinib 3d
tofacitinib 7d
Figure S2. Bar graph and representative dot plots show the percentage of splenic NK cells double positive for KLRG1 and CD11b after 3 and 7 days of tofacitinib treatment.

## Slide 3
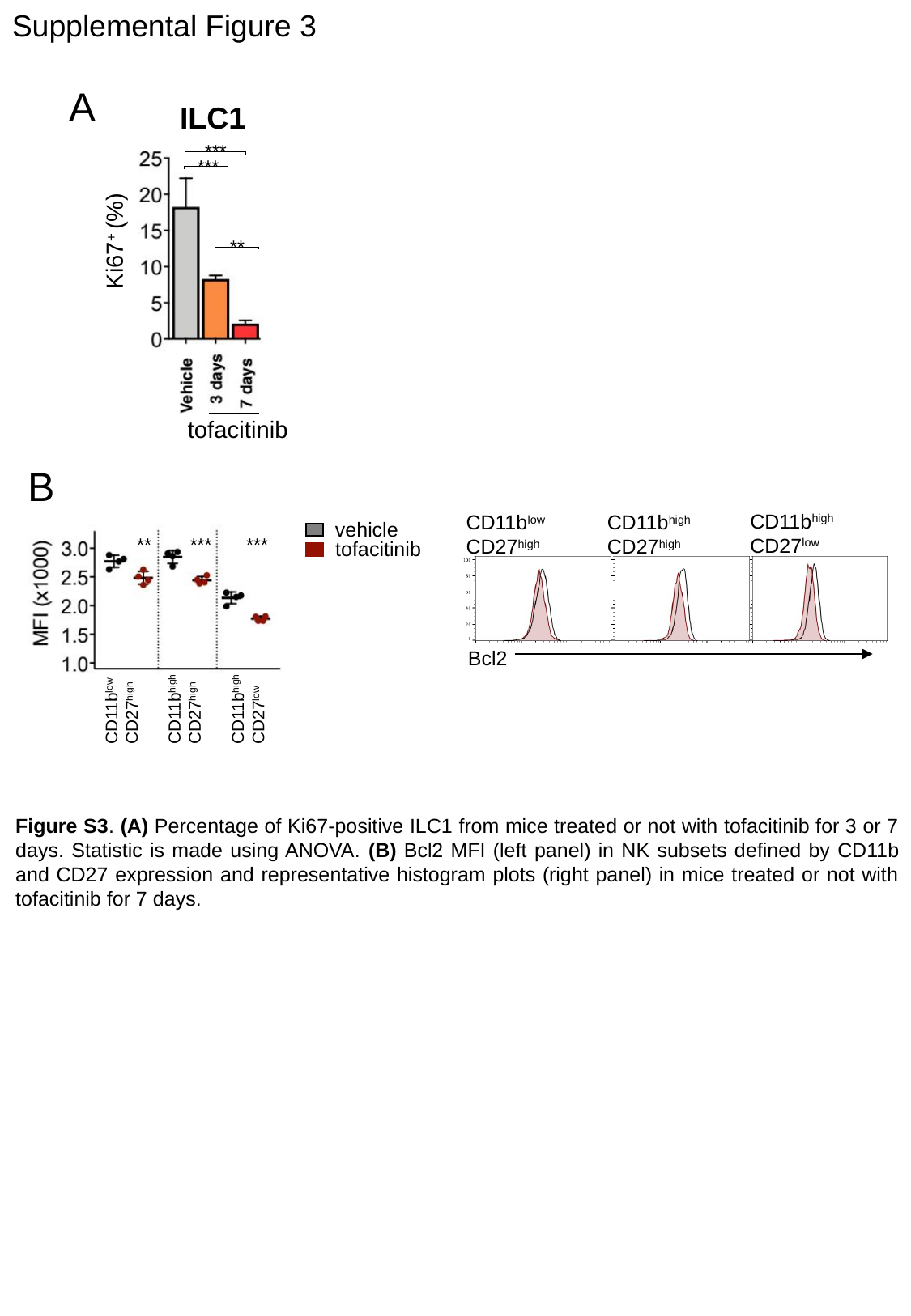

Supplemental Figure 3
A
ILC1
***
***
Ki67+ (%)
**
tofacitinib
B
CD11bhigh
CD27low
CD11bhigh
CD27high
CD11blow
CD27high
vehicle
tofacitinib
**
***
***
Bcl2
CD11blow
CD27high
CD11bhigh
CD27high
CD11bhigh
CD27low
Figure S3. (A) Percentage of Ki67-positive ILC1 from mice treated or not with tofacitinib for 3 or 7 days. Statistic is made using ANOVA. (B) Bcl2 MFI (left panel) in NK subsets defined by CD11b and CD27 expression and representative histogram plots (right panel) in mice treated or not with tofacitinib for 7 days.
